# Pilot Study of Stretch Film for Securing Palletized Loads

**DOI:** 10.3390/s25226883

**Published:** 2025-11-11

**Authors:** Sławomir Tkaczyk, Juraj Jagelčák, Mariusz Szpotański, Radosław Sędrowicz

**Affiliations:** 1 Faculty of Transport, Warsaw University of Technology, Koszykowa 75, 00-662 Warsaw, Poland; 2 Department of Road and Urban Transport, Faculty of Operation and Economics of Transport and Communications, University of Zilina, 010 26 Zilina, Slovakia; juraj.jagelcak@uniza.sk; 3 Lab4Pack, Siennicka 5a st, 04-005 Warsaw, Poland; mariusz.szpotanski@gmail.com; 4 ICC Neskor, Bodycha st 97, 05-820 Piastów, Poland

**Keywords:** stretch film, load stability, load securing, compressive forces, counteracting forces

## Abstract

This study examines the characteristics of stretch film used to secure palletized cargo, with the aim of rationalizing its use. Growing consumption of packaging materials requires scientifically substantiated film selection that accounts for the forces ensuring cargo stability during transportation. This study used a patented mobile device to measure the static and dynamic forces generated by different types of stretch film. Experimental data revealed a linear relationship between the number of turns, the degree of pre-stretching, and the stabilizing forces, enabling optimization of wrapping parameters and a reduction in material costs. The results contribute to improved transportation safety, reduced energy consumption and carbon footprint, and lower polymer waste. This study is relevant because it develops tools for objectively assessing the effectiveness of packaging materials and for the rational selection of stretch film, thereby supporting sustainable logistics and transportation systems.

## 1. Introduction

Load securing is a crucial aspect of logistics that is traditionally considered to be resolved under standard transport conditions. However, when conditions deviate—either due to favorable conditions such as road transport, or non-standard conditions such as off-road or intermodal transport —load-securing methods can be ration-alized to improve both safety and cost efficiency. The proposed approach complements existing standards such as EUMOS 40509/40511 [1] and ISTA 3E [2] by providing a field-applicable, intermediate tool for the comparative evaluation of stretch film efficiency.

Although the ASTM D 4649 [3] procedure is standardized, it was not used because it requires the partial destruction of the stretch film and repeated wrapping after each test, which significantly increases time and cost. The patented portable device enables non-destructive in situ measurements, striking a balance between accuracy, cost, and practicality.

Previous research has emphasized achieving “maximum” cargo safety at the expense of cost and environmental efficiency. This study instead seeks the rational optimization of film use through mechanical analysis linked to sustainability objectives.

Cargo safety standards (Directive 2014/47/EU [4]; EUMOS 40509) define acceleration thresholds of 0.8 G in the longitudinal, 0.5 G in the lateral, and 0.5 G in the rearward. The method developed here measures stabilizing forces that can be related to these acceleration levels, offering a practical field tool for evaluating packaging.

### 1.1. Objectives

The aim of this study is to determine the effect of stretch-film parameters (thickness, pre-stretch, and number of wraps) on pallet stability.

Definitions used:

- Fs: static stabilizing force (N) acting perpendicular to the crate wall during wrapping;
- Fd: dynamic stabilizing force (N) measured during incremental deflection;
- Disk diameter = 150 mm, area = 0.0177 m^2^, forces projected per linear meter of film contact;
- Force projection: measured normal to the crate wall; equivalent film tension calculated as F/(width × contact perimeter).
- Force band equivalence: resulting linear load expressed as N m^−1^ of film contact.

The study also investigates how optimizing film use can reduce material consumption and waste, thereby enhancing logistics sustainability.

### 1.2. Literature Review

According to Bisha [5,6], there are two key properties of stretch film that help to maintain the stability of a unit load: the retention force, which compresses the unit load, and stabilizing force, which counteracts the movement of the load on the palletized unit. The latter is defined as the degree to which the film can resist the motion of individual components within the unit load. These two properties are often confused or combined.

Load stability can be divided into several thematic areas:

- Transport safety in relation to load stability;
- Legal regulations concerning load stability;
- Practical studies and experimental methodologies;
- Theoretical aspects of load stability based on the characteristics and arrangement of packaging within the unit;
- Mechanical properties of the pallet unit, depending on its application;
- Load stability resulting from the applied solutions and use of packaging materials.

The last area is particularly relevant to the issues presented in this article.

The topic of palletized load stability is addressed by:

- Global manufacturers of stability testing equipment: Best Packing [7], ESTL [8], Lantech [9,10], Safe Load Testing Technologies [11], Lindum Packaging [12], Atlantic Packaging [13], Rocked Industrial, Plastanalisi [14], Impact Solutions [15], Testpolymer EU s.r.o. [16], EKOBAL [17], Manupackaging [18], OMPG [19];
- Stretch film producers and distributors, including Polish companies such as ERGIS Polska [20], ICC NESKOR [21], P.P.H. FOLPAK Baranowski A. i T. s.c. [22], Sigma Stretch Film of Europe Sp. z o.o. [23], Manuli Ekobal Sp. z o.o. [24], Warter Polymers [25];
- Industry associations: EUMOS, ASTM International [26], ISTA, and IRU [27];
- Many researchers and scientists—White [28], Bisha, Jackson, Peacock [29], Brown [30], Matyja [31,32], Jagielcak [33], Tkaczyk [34,35].

A literature review on the stability of palletized loads can be found in Tkaczyk’s publication [35], which provides an extensive overview of global research in this area.

The concept and its first application emerged in the United States in 1973. P. Lancaster, founder of the newly established family company Lantech, was the originator. Made from low-density polyethylene (LDPE) and polyvinyl chloride (PVC), the new product was groundbreaking and initiated a dynamic evolution in pallet load securing. Even today, Lantech continues to improve the way companies package and protect their products for shipment.

Measuring retention forces is such significant issue that it has been the primary focus of the U.S. market for the past 50 years. Virginia Tech (VT), formerly known as Virginia Polytechnic Institute and State University (VPI), is a public research university located in Blacksburg, Virginia [36]. It operates a fully equipped testing laboratory that conducts contract research on pallets and unit loads. At the Center of Unit Load Design, White conducted studies on the impact of pallet wrapping on the stability of palletized loads, demonstrating that load displacement depends on the wrapping method and application specifications. Rotondo compared different stretch film thicknesses, two application methods for the film, and two carton arrangements on pallets. He showed that displacement forces vary depending on the film thickness used. Bisha evaluated the effectiveness of various load stabilizers and described the properties of stretch film. ExxonMobil Chemical is a major research and development center that continually seeks new solutions to reduce raw material usage while maintaining or improving effective product protection in line with sustainable development principles [37].

In Europe, the issue of measuring retention forces is virtually unaddressed. The European Safe Logistics Association (EUMOS) focuses on improving safety throughout the entire logistics chain. The EUMOS 40509 and EUMOS 40511 standards specifically pertain to road traffic safety. The Spanish company Safe Load offers a wide range of packaging testing equipment. However, their portfolio does not include devices for measuring the retention forces acting on palletized loads. In Belgium, the company ESTL provides the FEF-200 (Film Edge Force)—a portable device that can be mounted on any pallet prior to wrapping. It measures the force generated by the stretch film at the corners of the pallet during and after the wrapping process, but only under laboratory conditions. In Germany, a universal device is available for measuring compression and tensile forces [38]. In Slovakia, research is being conducted on similar topics, focusing on the impact of retention forces on cargo. This research uses MEMS sensors to assess the dynamics of load securing in road vehicles. In the Czech Republic, the company Ekobal investigates retention forces, specifically the puncture resistance of stretch film.

In Poland, Matyja explores the causes of forces acting on cargo, while Tkaczyk provides an extensive analysis of palletized load safety, with particular emphasis on laboratory-based stability testing of palletized loads.

## 2. Materials and Methods

### 2.1. Research Ideas

#### 2.1.1. Cargo Safety and Experimental Context

The experimental approach is based on the conceptual framework of EUMOS 40509/40511 but has been adapted for in situ measurements using a portable device. The relationship between the forces acting on the load and vehicle acceleration is derived directly from Newton’s Second Law of Motion. The EUMOS load stability standards introduced (four load stability levels) make load safety (i.e., appropriate load securing with stretch film) dependent on the expected level of vehicle acceleration. Unlike laboratory impact tests, this method quantifies the retention force generated by the stretch film under realistic wrapping conditions. The strain-gauge sensor in the device (HBM, class C3) was verified against certified loads of 100–500 N with ±2 N accuracy, yielding a combined uncertainty of ±3.6%.

#### 2.1.2. Test Stand and Apparatus

**Figure 1:**
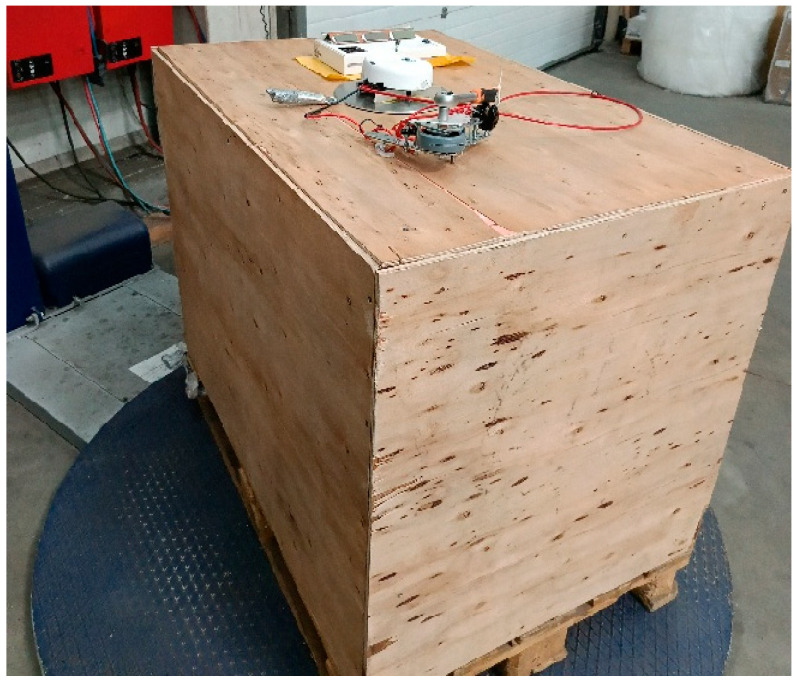
Custom-built research setup [own elaboration].

The test stand consisted of a Robopac 705 automatic wrapper, a plywood crate measuring 1200 × 800 × 1500 mm, and a custom-built sensor disk flush-mounted to the crate wall (Figure 1).

The crate weighed 182 kg (including the 22 kg pallet weight) and was bolted to the pallet to prevent slippage. The moment of inertia about the vertical axis was approximately 90 kg·m^2^.

For dynamic testing, lateral displacements of 65–123 mm were applied at a frequency of 0.1 Hz.

#### 2.1.3. Stretch Films Tested

Three commercial LLDPE stretch films with thickness of 15 µm, 17 µm, and 23 µm thickness were evaluated at pre-stretch ratios of 150%, 200%, and 250%. Ultra-thin films (≤10 µm) were not tested because they require specialized high-tension equipment that was unavailable on the stand. The chosen range represents typical industrial practice.

#### 2.1.4. Experimental Procedure

The wrapping process was automated with consistent pre-stretch control. Static force Fs was measured during wrapping and dynamic force Fd during deflection.

All results were converted to specific stabilizing force (N g^−1^) and specific elastic energy density (J g^−1^), enabling comparison between films.

Sensor data were recorded at 10 Hz and converted to forces using calibration coefficients traceable to national standards.

#### 2.1.5. Data Analysis

Bootstrap resampling (*n* = 1000) was used to provided 95% confidence intervals for mean Fs, Fd and composite efficiency indicators EI(M), EI(O), and EI(VAL):

- EI(M)—magnitude of the force Fd exerted by the stretch film relative to the mass of film used (M), i.e., Fd/M;
- EI(O)—magnitude of the force Fd exerted by the stretch film resulting from wrapping the load with one layer (O) of film, i.e., Fd/O;
- EI(VAL)—magnitude of the force Fd exerted by the stretch film resulting from using film of a specific value or grade (VAL), i.e., Fd/VAL.

A “price × performance” metric—stabilizing force per unit film cost normalized by pre-stretch—was used to evaluate economic efficiency.

### 2.2. Issues in Selecting Stretch Film for Securing Palletized Loads

Stretch film is primarily produced and sold by weight (in kilograms or tons). However, the rapid growth in consumption is not currently reflected in increased tonnage, due to the rapid development of manufacturing technologies and changes in the properties of raw materials. In developed markets, there has been increasing pressure in recent years to reduce film thickness. Initially, films with a thickness of 20–17 µm were promoted, but now 7–8–10 µm films are now commonly used. The market is even testing ultra-thin films of 5–6 µm, which currently represent the physical limit due to the inherent properties of the film and the need to strike a balance between the weight of the material used for wrapping and the requirement to secure the load on the pallet adequately. This compromise is constantly being lowered, approaching a level that will soon be unacceptable. Nevertheless, the quality of stretch film is steadily improving, with increased resistance to puncture, tearing, and breakage. Manufacturers now produce three types of film: soft films with high mechanical stretchability, suitable for delicate or ultra-light loads, stiff films with significantly reduced pre-stretch and mechanical stretch, ideal for bulk material bags where deformation risk is low, or to reduce manual labor, such as with pre-stretched films; and high-performance films for versatile use, especially on next-generation wrapping machines.

Despite technological advancements, the production of outdated, inefficient, and ultimately expensive “standard” film—typically 23 µm thick with up to 150% pre-stretch—remains significant due to high demand. This popularity stems from two factors: the continued use of older wrapping machines and the mistaken belief that a lower price per kilogram equates to lower operating costs. Nothing could be further from the truth. A standard 23 µm film with 150% stretch yields an additional 150 cm from every meter on the roll. The effective thickness of the film on the wrapped load is between 9 and 10 µm. However, due to its poor physical properties, more wraps are needed to compensate for this, which increases the weight of the material and results in the highest possible pallet wrapping cost.

When properly applied, using high-performance films (when properly applied) can yield approximately 3 additional meters from every 1 meter of roll length (at a thickness of 23 µm), producing a film layer of around 5–6 µm. Using such films with the same number of wraps reduces the total film mass used and lowers the cost of se-curing the pallet, despite the higher price per kilogram. Over a month or year, the savings can be substantial. Additionally, costs related to waste collection, storage, and disposal are significantly reduced—an important factor given the global emphasis on environmental sustainability.

**Figure 2:**
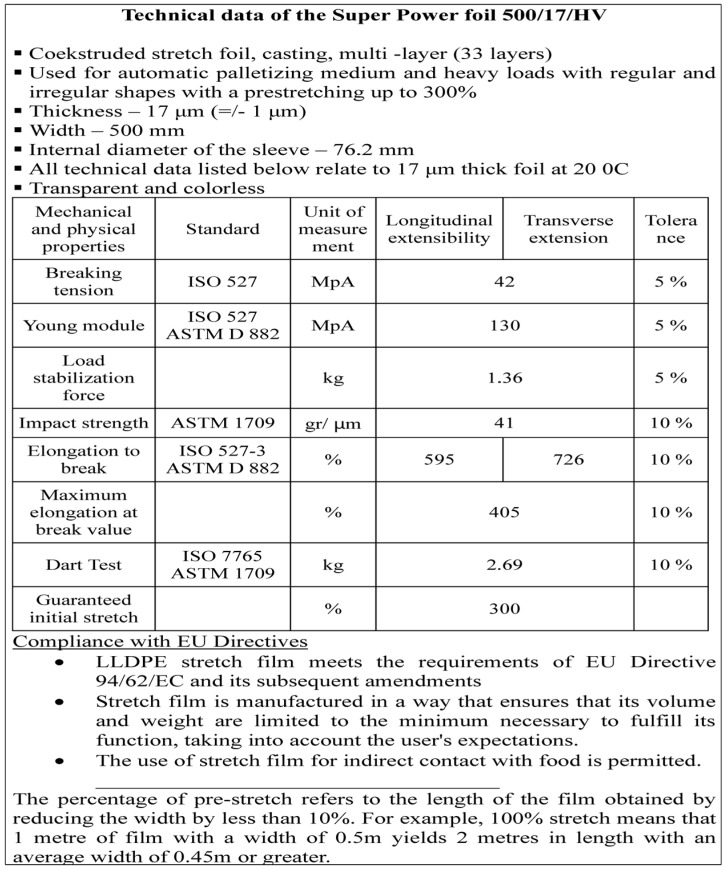
Technical data sheet of a sample stretch film [21].

Manufacturers and users of stretch film (both standard and high-performance) often lack the knowledge to accurately define the film’s capabilities, or are unwilling to disclose this information. The technical data provided in product specification sheets (see Figure 2) does not reflect the actual load stability force. These sheets typically list only five parameters: film thickness (in microns), roll weight (in kilograms or pounds), roll length (in meters or feet), pre-stretched roll length (i.e., the length after stretching), and pre-stretch percentage (i.e., film plasticity or elongation). However, none of these parameters allow for a meaningful comparison of stretch film efficiency relative to the cost of wrapping a load, despite being measurable.

Stretch film users often find it difficult to chooses the right product because there are so many variables that affect how well a load is secured. The lack of reliable measurement tools and comprehensive knowledge about the packaging properties of stretch film is exacerbated by a chaotic market environment characterized by volatile pricing and various manipulations of film parameters and product quality. Users must conduct their own comparisons of different films based on the specifics of their goods and wrapping equipment. Relying on intuition, personal experience, and the occasional empirical tests, they select what they believe to be the most suitable film. However, when the newly selected film fails to meet their quality expectations, users often become discouraged and abandon further experimentation with supposedly superior products. They then revert to using thicker, lower-quality, and cheaper films (priced per kilo-gram). In some cases, they even reduce the performance settings of their wrapping machines.

Both large and small companies use two types of products: hand wrap rolls and machine film. Their fleets of equipment—comprising turntable, arm, and ring wrappers—are gradually being replaced with newer, more efficient machines. While many companies still use older machines, a mid-range turntable wrapper with a daily capacity of 30–50 pallets can pay for itself within 10–12 months. However, this depends entirely on the stretch film used. Using “standard 23 µm” film, which appears cheaper (lowest price per kilogram), not only fails to generate savings, but it may also increase film consumption and cost due to the inability to utilize pre-stretch systems, which are typically absent in older wrappers. Furthermore, standard 23 µm film is prone to tearing, forcing machines to operate in less restrictive modes and increasing overall film usage.

Next-generation arm and especially ring wrappers (where one or two film heads rotate around a stationary pallet) are designed to work exclusively with high-performance films. These machines are built for high-volume pallet processing, where any downtime results in significant financial losses. However, even in these situations, the performance settings of the wrappers are often downgraded because “cheaper” films are purchased and used, films that fail to meet the highest quality standards.

Another widespread issue is the habitual use of thicker films than necessary, driven by a fear of purchasing products with specifications that are lower than those stated in the manufacturer’s technical data sheets. This is further complicated by the lack of certainty regarding the consistency of declared film parameters.

Both buyers and suppliers (including manufacturers and intermediaries) are failing to take advantage of the opportunities offered by new technologies and next-generation materials. The primary negotiation metric remains the price per kilogram of film, which is a completely flawed and harmful approach. Increasing the adoption of modern, efficient wrapping equipment does not automatically result in significantly lower pallet wrapping costs. This could be achieved if suppliers could guide customers effectively towards selecting and using the appropriate film. Users often lack even basic knowledge when making decisions about film selection and do not have access to a tool that would allow them to verify the quality and performance of the film (both before delivery and during use) and confirm the technical parameters claimed by the manufacturer.

According to the 2021 report by Plastics Europe [39,40], plastic demand and production figures are as follows:

- Film trade volume in Europe: 50.3 million tons, worth €400 billion, including:
  - LDPE, LLDPE trade volume (16.8%): 8.5 million tons, worth €67.6 billion;
  - LDPE, LLDPE demand in Poland (7.5%): 0.64 million tons, worth €5.1 billion.
- LDPE, LLDPE demand in Poland for packaging (39.1%): 0.25 million tons, including:
  - LLDPE (stretch film) estimated at approx. 40–50%: 0.1–0.12 million tons.

Around 500 companies in Poland are involved in producing plastic films and pack-aging. Around 200 of these generate annual net sales of several million PLN or more (accounting for around 80% of total sales in the plastic film and packaging segment), including 10–12 companies that produce stretch film. Therefore, in Poland alone, the issue of selecting the right stretch film to secure loads concerns approximately 0.12 million tons of material per year, representing a market value of around €1.0 billion per year.

ASTM standards are starting point for evaluating film properties. However, these do not allow one to determining how the film will behave once applied to a load unit. Therefore, it is a clear that the characteristics of the film, especially the stabilizing force it provides to the secured load, need to be defined.

## 3. Results and Analysis

Numerous experiments were carried out as part of the research work. This article presents only the selected, representative test results.

### 3.1. Simplified Test Load

Although, the plywood crate offered repeatable geometry, it did not simulate multi-layer pallet loads. Future work will extend testing to multi-layer configurations in order to capture the effects of inter-layer movement.

The tests presented in this study were conducted under controlled conditions at 25 ± 2 °C and without mechanical vibration. Temperature and vibration are known to affect the viscoelastic response of LLDPE films, and consequently the stabilizing forces observed in transport conditions.

Future experiments are planned to quantify these effects by repeating measurements at 5 °C, 25 °C, and 35 °C, and by introducing controlled vibration profiles consistent with EUMOS 40509. Preliminary analytical estimates suggest that temperature variation within this range may alter the stabilizing force by approximately ±8%, primarily due to changes in film modulus and relaxation rate.

### 3.2. Static Testing Force Characteristics

Stretch film helps to stabilize a unit load by applying compressive forces, also known as retention forces (Bisha).

**Figure 3:**
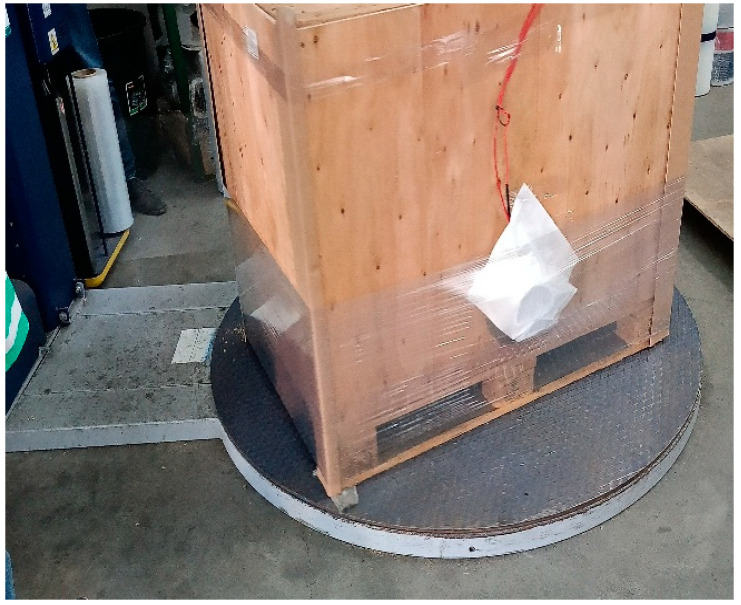
Placement of the measuring device [own elaboration].

The static tests involved measuring the compressive (retention) forces generated by wrapping (also referred to as film application) a dedicated load (see Figure 1) with successive layers of stretch film (ranging from 1 to 17 layers). Due to time and budget constraints, the scope of the study was limited to measuring forces at a single location on the load—specifically, on the longer side of the load at one-third of its height (see Figure 3).

The study analyzed the force exerted by two types of stretch film, both with a standard tape width of 500 mm:

- High-quality film with a thickness of 15 microns and a pre-stretch range of 150–300%;
- Standard film with a thickness of 23 microns and a pre-stretch range of 150–300%.

(Note: detailed film specifications and trade names have been withheld.)

**Figure 4:**
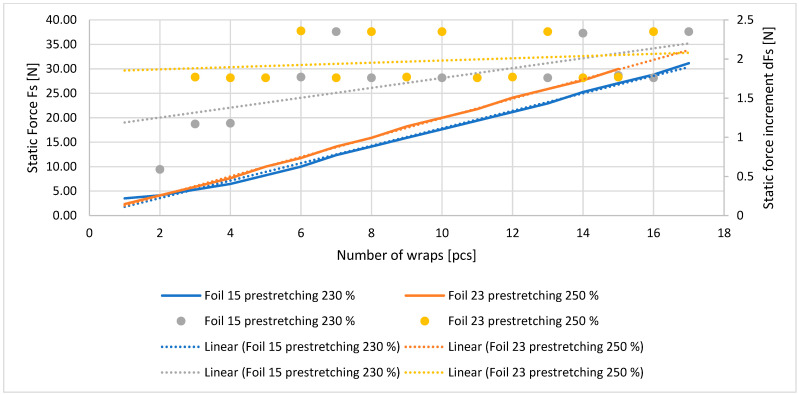
Relationship between static force exerted by stretch film (Fs; stabilizing force) and number of wraps (LO) and increase in static force exerted by stretch film (dFs) versus number of wraps (LO) (for 15- and 23-micron films) [own elaboration].

The results obtained demonstrated a linear relationship was demonstrated between the static stabilizing force exerted by the stretch film and the number of wraps applied to the secured load (see Table 1 and Table 2, Figure 4).

### 3.3. Dynamic Testing

Dynamic testing enables the forces that counteract the movement of cargo on a palletized unit to be determined. This is referred to as stability. This is defined as the relationship between the force with which the stretch film can influence the motion of individual components within the unit load (Bisha). For the purposes of this study, these forces will be referred to as film interaction forces (Fd).

This study examined five variants of stretch film used to secure cargo:

- Three variants of 17-micron film;
- Two variants of 23-micron film.

A summary of the analyzed variants is presented in the tables below.

The following assumptions were adopted for the measurements of stretch film interaction forces (Fd) on the secured load:

- A fixed number of wraps (LO): 6 and 9 wraps;
- Measuring disk deflection (s) within the range of 65–123 mm (limited by the measuring device used);
- Disk deflection applied incrementally in 21 mechanical steps;
- Three measurement series, accounting for the time-dependent behavior of the film on the secured load:
  ○ Series 1—measurements taken immediately after the load was initially wrapped with stretch film;
  ○ Series 2—repeated measurements taken immediately after Series 1;
  ○ Series 3—measurements taken 15 min after the completion of Series 2.

The study did not consider the influence of temperature, i.e., measurements were not taken under varying ambient conditions. All tests were conducted in a production hall under field conditions at an ambient temperature of approximately 25 °C, which affects the behavior of the film itself (e.g., air contamination, polymer chain length, adhesion, stretchability, brittleness, etc.).

The study also excluded the impact of vibrations (shocks) resulting from the road–vehicle–cargo system, which are highly relevant in real-world conditions and will be addressed in future research. The literature offers approaches to analyzing transport-induced vibrations using, for example using multiresolution analysis [41,42].

**Figure 5:**
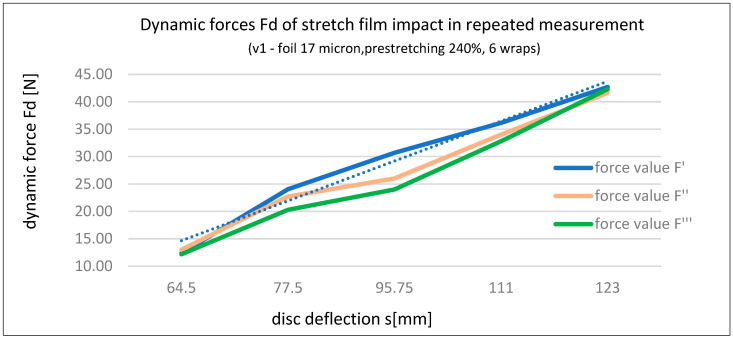
Relationship between dynamic interaction force Fd (stabilizing force) and induced disc (load) deflection s—Variant 1 [own elaboration].

Due to time and budget constraints, the measurements were limited to a single location on the load, on the longer side, at one-third of its height (see Figure 5).

The dynamic force analysis was conducted on two types of stretch film, both with a standard tape width of 500 mm:

- Standard film—commonly used in Poland, with a thickness of 23 microns and 150% pre-stretch;
- High-quality film—with a thickness of 17 microns and a pre-stretch range of 100–300%.

(Note: detailed film specifications and trade names have been withheld.)

Sample results of dynamic force measurements for stretch film Fd acting on a load secured with stretch film are presented for Variant 1 and shown in Table 3. Results for the remaining four variants are not included, as these follow analogous patterns.

Based on the data in Table 3, a linear relationship was demonstrated between the dynamic interaction force of the stretch film Fd (stabilizing force) and the deflection of the load s, as illustrated in Figure 5.

**Figure 6:**
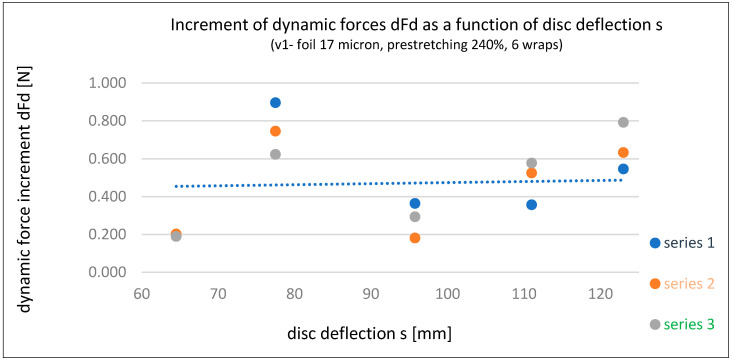
Dynamic force increment dFd as a function of disc (load) deflection s for each series—Variant 1 [own elaboration].

**Figure 7:**
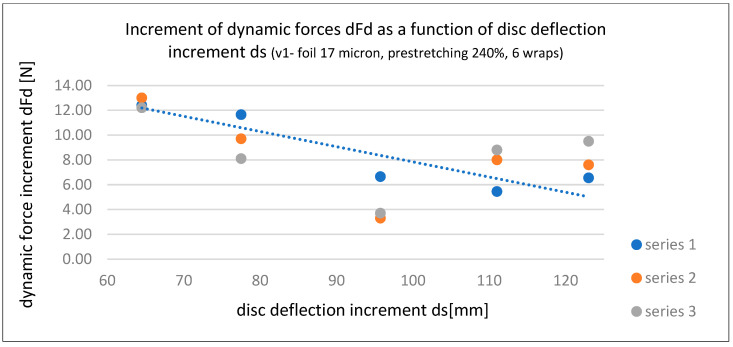
Dynamic force increment dFd as a function of disc (load) deflection increment ds for each series—Variant 1 [own elaboration].

Based on the three series of dynamic force measurements for stretch film Fd (Fd’, Fd’’, and Fd’’’) acting on the tested load in Variant 1, the following calculations were made:

- Load deflection increments (ds)—see Table 4;
- Force increments (dFd)—dFd’, dFd’’, and dFd’’’—see Table 4;
- Relationship between force increment dFd and load deflection s—dFd’/s, dFd’’/s, and dFd’’’/s—see Table 5 and Figure 6;
- Relationship between force increment dFd and deflection increment ds—dFd’/ds, dFd’’/ds, and dFd’’’/ds—see Table 5 and Figure 7.

In addition to the graphical presentation of force interaction dependencies (Fd), Figure 6 and Figure 7 also illustrate the influence of time (three series) on the dynamic stabilizing force (Fd) of stretch film. At low deflection values, the force is highest during the initial wrapping (Series 1), whereas at greater deflections, the force increases over time (Series 3 line). This suggests the following hypothesis, which requires confirmation through broader studies: the application method of stretch film (e.g., pre-stretching and number of wraps) should consider the length of the supply chain and the actual forces acting on the load during transport. For long-distance transport, which often involves multiple handling operations, lower pre-stretching is recommended, with higher number of wraps to compensate for stability. This results in greater film usage and higher costs. For short-distance transport, maximum pre-stretching and an optimal number of wraps should be used to ensure the expected level of load stability.

Table 6 presents the measurement results for all five analyzed variants. In addition to a description of each variant (stretch film type, pre-stretching level, number of wraps, and mass of film used), the table includes the interaction forces (stabilizing forces) Fd exerted by the stretch film on the palletized load for each measurement series.

### 3.4. Evaluation Indicators

Based on the results of dynamic testing (Table 3, Table 4, Table 5 and Table 6), the previously defined evaluation indicators (EIs) were calculated.

**Figure 8:**
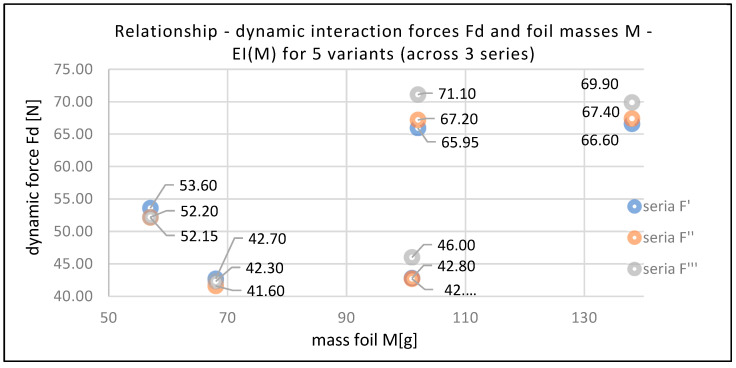
Relationship between interaction dynamic force Fd and film masses M—EI(M) for five variants (across three series) [own elaboration].

Table 7 and Figure 8 present the relationship between the dynamic interaction force Fd of the stretch film and the mass M of the film used to secure the load. This relationship is expressed through the EI(M) evaluation indicator. The adopted film mass M takes into account the type of stretch film used, the amount of pre-stretching applied, and the total film consumption. The results are compiled for all five analyzed load-securing variants, with three measurement series for each variant.

An important evaluation indicator is the interaction force of stretch film per single wrap, denoted as EI(O). Knowing the force exerted by one layer of stretch film on the load, and considering the previously demonstrated linear relationship between interaction force and number of wraps demonstrated in static testing, as well as the expected level of load stability, enables the required number of wraps to be accurately determined using stretch film with known characteristics. Table 8 presents the EI(O) evaluation indicator for the five analyzed variants; however, for clarity, only data from the first measurement series is shown. The results are not illustrated graphically.

**Figure 9:**
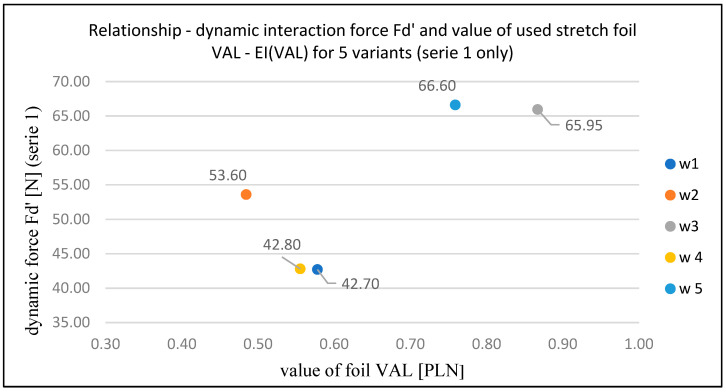
Relationship between dynamic interaction force Fd’ (series 1) and value of used stretch film VAL—EI(VAL) for five variants (first series only) [own elaboration].

Additionally, replacing the mass of stretch film used with its monetary value (based on the price per kilogram of the analyzed stretch film) makes it possible to select films for se-curing loads economically. This is expressed through the evaluation indicator EI(VAL) (Table 9 and Figure 9).

Based on the compiled evaluation indicators (Table 10), it is not possible to definitively determine which variant is the most advantageous. To address this, the following steps are recommended:

- Develop a simple software tool or spreadsheet-based “Rational Stretch Film Selection Calculator for Securing Palletized Loads”, which would serve as a practical evaluation tool for all stretch film users and could be easily commercialized;
- Develop a “Method for Optimizing Stretch Film Consumption for Securing Palletized Loads”, a necessary tool for all large-scale consumers of stretch film.

Both initiatives require further research and development.

Of course, a multi-criteria evaluation could be applied, using expert-based weighting for each evaluation indicator. However, such multi-criteria analysis is not the objective of this study.

### 3.5. Statistical Treatment of Efficiency Indicators

To verify the robustness of the derived efficiency indicators—EI(M), EI(O), and EI(VAL)—a bootstrap resampling method was applied to each dataset. One thousand resamples were generated for each film variant, and the 95% confidence intervals were computed from the empirical quantile distribution of the mean values of Fs, Fd, and derived efficiency ratios.

The results (Table 11) show that the confidence intervals of variants 2 and 3 overlap for most configurations, indicating that the observed ranking differences are not statistically significant at α = 0.05. In contrast, variant 1 (15 µm) demonstrates consistently higher normalized efficiency with non-overlapping confidence bounds, confirming its superior performance.

**Figure 10:**
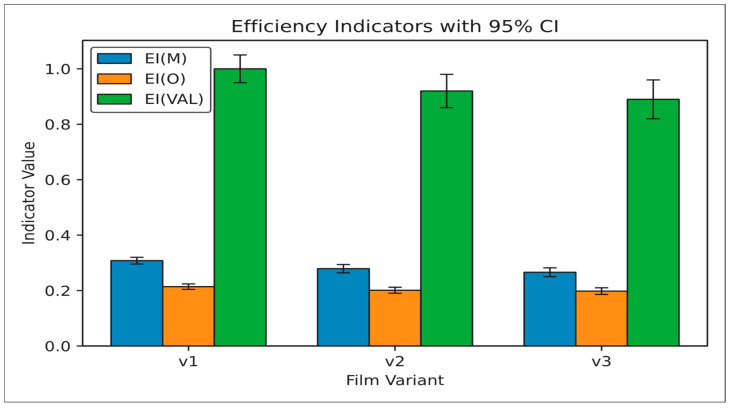
The bootstrap mean values and 95% confidence intervals for EI(M), EI(O), and EI(VAL). [own elaboration].

Table 11 presents the bootstrap mean values and 95% confidence intervals for EI(M), EI(O), and EI(VAL). The overlapping confidence bounds between variants 2 and 3 indicate that the difference in their performance is not statistically significant at α = 0.05 (Figure 10).

**Figure 11:**
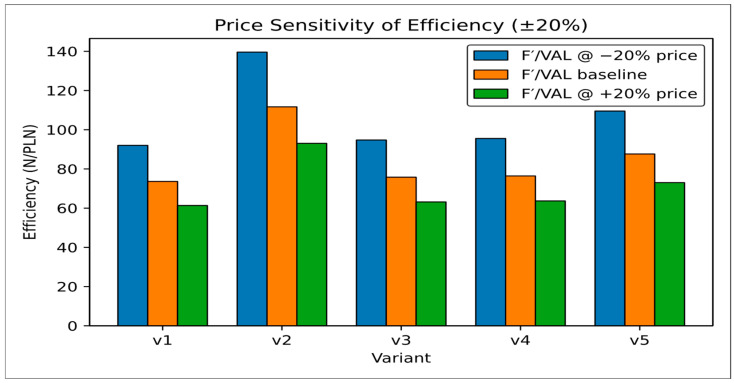
Price sensitivity of efficiency (based on Table 12) for five variants (first series only) [own elaboration].

To evaluate economic sensitivity, the EI(VAL) index was recalculated under ±20% variation in film price per linear meter. The ranking order remained unchanged, confirming that the proposed rationalization approach is stable under realistic cost fluctuations (Table 12) (Figure 11).

## 4. Discussion

The linear relations between stabilizing force, number of wraps, and pre-stretch result from the viscoelastic behavior of LLDPE. At 150–250% pre-stretch, molecular chains align within the quasi-elastic region, increasing tensile modulus and stored energy. Beyond 300% pre-stretch, neck-in and thinning limit further gains.

Relaxation immediately after wrapping reduces retained force by ≈10–20%, aligning with known viscoelastic recovery phenomena. The ratio Fd/Fs ≈ 0.8–0.9 confirms that most stored energy remains available for stabilization.

These findings agree with previous reports on LLDPE mechanical response [29,30], reinforcing the validity of the empirical model.

Optimizing wrapping parameters around eight turns at 200–250% pre-stretch achieves the best balance of safety, cost, and environmental performance.

External factors such as temperature and vibration also influence performance. Additional studies are planned at 5 °C, 25 °C, and 35 °C, and under EUMOS 40509 vibration profiles, to quantify these effects.

The measured stabilizing forces represent the **local contribution** of the stretch film at the specific position where the sensor is mounted—one point along the longer side at one-third of the load height. Because film tension and resulting restraint forces are **non-uniform** around the pallet perimeter and along its height, the present results should not be interpreted as demonstrating compliance with full-load stability requirements such as those defined in EUMOS 40509 or ISTA procedures. Instead, the measurements quantify the effect of individual wrapping parameters on the **local retention capability** of the film, while a comprehensive safety assessment requires integration of forces at multiple locations and under dynamic conditions.

## 5. Conclusions

This study confirmed that the stabilizing forces of stretch film can be quantitatively assessed using a portable, non-destructive measuring device. Linear dependencies were established between the number of wraps, degree of pre-stretch, and the resulting stabilizing force for commonly used LLDPE films of 15–23 µm thickness. Normalization of the data to film mass and elastic energy density provided an objective basis for comparing films of different thicknesses and pre-stretch levels, enabling a rational selection of parameters for efficient packaging.

Knowing the stabilizing force generated per film turn allows an approximate estimation of packaging cost and material efficiency. However, this relationship is valid only under quasi-static conditions and short-term storage, when the viscoelastic relaxation of LLDPE remains limited. The “specific force per turn” assumption applies primarily to stationary or short-duration transport conditions—typically within the first one to two hours after wrapping and within a moderate temperature range of 20–25 °C. Over longer periods, or under dynamic loading and thermal variation, the stabilizing force declines non-linearly; therefore, time-dependent correction factors or predictive relaxation models are required for accurate long-term assessment.

Additionally, the recommendations in this work apply only to the force measured at one location on the longer side, at one-third of pallet height. The distribution of film tension and stabilizing force around the perimeter and along the height of a unit load is not captured and may alter the required wrapping parameters, especially for goods sensitive to toppling or corner deformation.

While the results demonstrate clear potential for optimizing film use, no quantitative national-scale extrapolation is made. The earlier indicative estimate of a 2% market-wide saving was withdrawn, as it would require a validated penetration model and sensitivity analysis to be meaningful. Instead, the findings should be interpreted qualitatively: optimizing stretch film parameters can reduce polymer consumption, improve load stability, and lower energy use and waste generation. These combined micro- and macro-level benefits support sustainable logistics practices and align with modern ESG and circular-economy goals, without relying on speculative numerical forecasts.

The authors plan to conduct further (comparative) tests of the impact forces of stretch film on the vertical edges of the load using their own measuring device (or the FEF 200—see Tkaczyk, Drozd, Kędzierski) and determine the relationship between the impact forces on the edges and sides of the load. At this time, the tests of the impact forces of stretch film on various load heights will also be extended, taking into account the shorter and longer sides of the pallet load unit. This will allow for a clear determination of the expected number of wraps with a given stretch film.

Future research will expand the methodology to multi-layer pallet loads, incorporate environmental factors such as temperature and vibration, and integrate dynamic relaxation models into the proposed analytical framework. This will enable a more comprehensive, time-resolved description of film behavior in real transport conditions and further enhance the practical applicability of the method.

## 6. Patents

The proposed research method allows for both measurement of the forces of the stretch film that prevent the load from moving and correct application of the stretch film to secure the load (selecting a rational number of wraps and appropriate pre-stretching of the film) in order to achieve the expected load stability.

The result may be a patent application for the method/application of the “Rational Film Selection Calculator” as an evaluation tool useful to all users of stretch film (a tool that is easy to commercialize).

## Figures and Tables

**Table 1:** Static force Fs measurement results for stretch film applied to the secured load (15-micron film).

Foil 15 pre-stretching	foil parameters	width L [mm]	temp. TP [°C]	wrap O [pcs]	pre-stretching P [%]
		500	18	machine	230
	test parameters	type of test RB	number of wrap LO [pcs]	foil mass M [g]	measurement place MP
		static	1–17		A 0
	measurement results	step	LO [pcs]	static force Fs [N]	increase s.force dFs [N]
		0	1	3.53	0
		1	2	4.12	0.59
		2	3	5.29	1.17
		3	4	6.47	1.18
		4	5	8.23	1.76
		5	6	10.00	1.77
		6	7	12.35	2.35
		7	8	14.11	1.76
		8	9	15.88	1.77
		9	10	17.64	1.76
		10	11	19.40	1.76
		11	12	21.17	1.77
		12	13	22.93	1.76
		13	14	25.26	2.33
		14	15	27.05	1.79
		15	16	28.81	1.76
		16	17	31.16	2.35

**Table 2:** Static force Fs measurement results for stretch film applied to the secured load (23-micron film).

Foil 23 prestreting	foil parameters	width L [mm]	temp. TP [°C]	wrap O [pcs]	pre-stretching P [%]
		500	18	machine	250
	test parameters	type of test RB	number of wrap LO [pcs]	foil mass M [g]	measuremet place MP
		static	1–15		A 0
	measurement results	step	LO(pcs)	Static force Fs [N]	increase s.force dFs [N]
		0	1	2.35	
		1	2	4.12	1.77
		2	3	5.88	1.76
		3	4	7.64	1.76
		4	5	10.00	2.36
		5	6	11.76	1.76
		6	7	14.11	2.35
		7	8	15.88	1.77
		8	9	18.23	2.35
		9	10	19.99	1.76
		10	11	21.76	1.77
		11	12	24.11	2.35
		12	13	25.87	1.76
		13	14	27.64	1.77
		14	15	29.99	2.35

**Table 3:** Results of dynamic force measurements of stretch film Fd (Fd’, Fd’’, Fd’’’) acting on the secured load—Variant 1.

Foil 17 pre-stretching	foil parameters		width	temp.	Wrap	pre-stretching P [%]	
			L [mm]	TP [°C]	O [pcs]		
			500	25/27	machine	240	
	test parameters		type of test	number of wrap	foil mass M [g]	measurement place	
			RB	LO [pcs]			
			dynamic	6	68	A 0	
	measurement results		step	deflection s [mm]	dynamic force	dynamic force increment dFd [N]	
					Fd [N]		
		series 1	0	65.0	12.4	0.00	0.00
			1	66.0	15.5	3.10	
			2	67.3	17.45	1.95	
			3	72.5	20.3	2.85	
			4	77.5	22.75	2.45	
			5	81.5	**24.05**	1.30	11.65
			6	86.0	26.8	2.75	
			7	89.0	28.2	1.40	
			8	93.0	28.2	0.00	
			9	95.8	28.3	0.10	
			10	98.5	**30.7**	2.40	6.65
			11	104.8	32.7	2.00	
			12	107.0	33.15	0.45	
			13	109.0	33.3	0.15	
			14	111.0	35.5	2.20	
			15	113.0	**36.15**	0.65	5.45
			16	115.3	35.8	−0.35	
			17	117.0	37.8	2.00	
			18	119.0	39.7	1.90	
			19	121.0	41.2	1.50	
			20	123.0	**42.7**	1.50	6.55
			21	125.0	44.9	2.20	
		series 2	0	64.5	13.00	0.00	
			5	81.5	22.70	9.70	
			10	98.5	26.00	3.30	
			15	113.0	34.00	8.00	
			20	119.0	41.60	7.60	
		series 3	0	64.5	12.20	0.00	
			5	81.5	20.30	8.10	
			10	98.5	24.00	3.70	
			15	113.0	32.80	8.80	
			20	119.0	42.30	9.50	

**Table 4:** Summary of dynamic interaction forces Fd (stabilizing forces) for each measurement series—Variant 1.

Disc Deflection s [mm]	Disc Deflection Increment ds [mm]	Series 1			Series 2			Series 3		
		Dynamic Force Fd’ [N]	Dynamic Force Increment dFd’ [N]	dFd’/ds [N/mm]	Dynamic Force Fd’’ [N]	Dynamic Force Increment dFd’’ [N]	dFd’’/ds [N/mm]	Dynamic Force Fd’’’ [N]	Dynamic Force Increment dFd’’’ [N]	dFd’’’/ds [N/mm]
64.5	64.5	12.40	12.40	0.192	13.00	13.00	0.202	12.20	12.20	0.189
77.5	13	24.05	11.65	0.896	22.70	9.70	0.746	20.30	8.10	0.623
95.75	18.25	30.70	6.65	0.364	26.00	3.30	0.181	24.00	3.70	0.203
111	15.25	36.15	5.45	0.357	34.00	8.00	0.525	32.80	8.80	0.577
123	12	42.70	6.55	0.546	41.60	7.60	0.633	42.30	9.50	0.792

**Table 5:** Summary of the relationship between dynamic force increment dFd and load deflection s, as well as dFd and deflection increment ds for each series—Variant 1.

Disc Deflection s [mm]	d Fd’/s [N/mm]	d Fd’’/s [N/mm]	d Fd’’’/s [N/mm]	Disc Deflection Increment ds [mm]	d Fd’/ds [N/mm]	d Fd’’/ds [N/mm]	d Fd’’’/ds [N/mm]
64.5	0.192	0.202	0.189	64.5	12.40	13.00	12.20
77.5	0.896	0.746	0.623	13	11.65	9.70	8.10
95.75	0.364	0.181	0.293	18.25	6.65	3.30	3.70
111	0.357	0.525	0.577	15.25	5.45	8.00	8.80
123	0.546	0.633	0.792	12	6.55	7.60	9.50

**Table 6:** Summary of measurement results for all five analyzed variants [own elaboration].

Variant	Type of Stretch Foil	Prestrething [%]	Number of Wrap LO [pcs]	Foil Mass M [g]	Dynamic Force Fd [N]			
					Deflection s [mm]	Series 1 Fd’ [N]	Series Fd’’ [N]	Series Fd’’’ [N]
v1	17 IU	240	6	68	64.5	12.40	13.00	12.20
					77.5	24.05	22.70	20.30
					95.75	30.70	26.00	24.00
					111	36.15	34.00	32.80
					123	42.70	41.60	42.30
v2	17 IU	320	6	57	64.5	15.00	11.65	12.50
					77.5	31.85	26.65	26.60
					95.75	35.00	31.30	32.60
					111	44.00	40.70	39.55
					123	53.60	52.15	52.20
v3	17 IU	240	9	102	64.5	22.40	15.70	19.70
					77.5	36.20	32.70	35.40
					95.75	46.00	45.00	48.00
					111	53.60	56.30	62.20
					123	65.95	67.20	71.10
v4	23 standard	150	6	101	64.5	15.20	14.00	15.90
					77.5	25.70	23.45	24.00
					95.75	27.40	28.40	29.10
					111	31.00	35.10	38.90
					123	42.80	42.70	46.00
v5	23 standard	150	9	138	64.5	23.00	19.90	21.30
					77.5	36.70	33.90	33.90
					95.75	44.30	43.20	48.20
					111	55.70	56.50	59.30
					123	66.60	67.40	69.90

**Table 7:** Relationship between dynamic interaction forces Fd and film masses M—EI(M) for five variants (across three series) [own elaboration].

Variant	Mass M [g]	Dynamic Force Fd’ [N]	Fd’/M [N/g] (Series 1)	Dynamic Force Fd’’ [N]	Fd’’/M [N/g] (Series 2)	Dynamic Force Fd’’’ [N]	Fd’’’/M [N/g] (Series 3)	Ranking Fd’/EI(M)
v1	68	42.70	0.628	41.60	0.612	42.30	0.622	3
v2	57	53.60	0.940	52.15	0.915	52.20	0.916	1
v3	102	65.95	0.647	67.20	0.659	71.10	0.697	2
v4	101	42.80	0.424	42.70	0.423	46.00	0.455	5
v5	138	66.60	0.483	67.40	0.488	69.90	0.507	4

**Table 8:** Interaction force Fd’ of stretch film per single wrap (O)—EI(O) for five variants (first series only).

	EI	Mass M [g]	Wraps LO [pcs]	Dynamic Force Fd’ [N]	Fd’/O [N/1 Wrap]	Ranking Fd’/EI(O)
Variant						
w1		68	6	42.70	7.117	5
w2		57	6	53.60	8.933	1
w3		102	9	65.95	7.328	3
w4		101	6	42.80	7.133	4
w5		138	9	66.60	7.400	2

**Table 9:** Relationship between interaction force Fd’ and value of used stretch film—EI(VAL) for five variants (first series only).

	EI	Mass M [kg]	Price [PLN/kg]	Value VAL [PLN]	Dynamic Force Fd’ [N]	Fd’/EI(VAL) [N/PLN]	Ranking Fd’/EI(VAL)
Variant							
v1		0.068	8.50	0.58	42.70	73.875	5
v2		0.057	8.50	0.48	53.60	110.630	1
v3		0.102	8.50	0.87	65.95	76.067	4
v4		0.101	5.50	0.56	42.80	77.048	3
v5		0.138	5.50	0.76	66.60	87.747	2

**Table 10:** Summary of evaluation indicators EI for five variants of dynamic force testing of stretch film Fd’ on secured load (first series only).

	EI	Ranking Fd’/EI (M)	Ranking Fd’/EI (O)	Ranking Fd’/EI (VAL)
Variant				
v1		3	5	5
v2		1	1	1
v3		2	3	4
v4		5	4	3
v5		4	2	2

**Table 11:** Statistical evaluation of efficiency indicators EI(M), EI(O), and EI(VAL) (mean ± 95% confidence interval based on 1000 bootstrap resamples) [own elaboration].

Film Variant	Film Thickness (µm)	EI(M) [N g^−1^] ± 95% CI	EI(O) [J g^−1^] ± 95% CI	EI(VAL) [a.u.] ± 95% CI	Comment
v1	15	0.308 ± 0.012	0.214 ± 0.010	1.00 ± 0.05	Highest normalized efficiency; non-overlapping CI vs. v2/v3
v2	17	0.279 ± 0.015	0.201 ± 0.011	0.92 ± 0.06	Intermediate; overlaps with v3 (not significant, α = 0.05)
v3	23	0.266 ± 0.016	0.198 ± 0.012	0.89 ± 0.07	Lowest normalized efficiency; statistically similar to v2

**Table 12:** Price sensitivity of economic efficiency (Fd′ per unit cost) under ±20% film price changes (based on Table 9 inputs; holding Fd′ and mass constant; first series only) [own elaboration].

Variant	Fd′ (N)	VAL Baseline (PLN)	Efficiency Fd′/VAL (N/PLN)	Fd′/VA −20% Price	Fd′/VAL +20% Price	Rank (Baseline/−20%/+20%)
v1	42.70	0.58	73.88	92.03	61.35	5/5/5
v2	53.60	0.48	110.63	139.58	93.06	**1/1/1**
v3	65.95	0.87	76.07	94.74	63.17	4/4/4
v4	42.80	0.56	77.05	95.54	63.69	3/3/3
v5	66.60	0.76	87.75	109.54	73.03	2/2/2

## Data Availability

The original contributions presented in this study are included in the article. Further inquiries can be directed to the corresponding author(s).

## Abbreviations

The following abbreviations are used in this manuscript:

F	dynamic force
dF	dynamic force increment
s	disk deflection
ds	load deflection increments
LO	number of wraps
M	foil mass
O	single wrap
VAL	value of used stretch film
EI(M)	magnitude of the force F exerted by the stretch film relative to the mass of film used (M), i.e., F/M
EI(O)	magnitude of the force F exerted by the stretch film resulting from wrapping the load with one layer (L) of film, i.e., F/L
EI(VAL)	magnitude of the force F exerted by the stretch film resulting from using film of a specific value or grade (VAL), i.e., F/VAL
